# *In vitro* and *in vivo* cytotoxic activity of human lactoferricin derived antitumor peptide R-DIM-P-LF11-334 on human malignant melanoma

**DOI:** 10.18632/oncotarget.17823

**Published:** 2017-05-11

**Authors:** Sabrina Riedl, Beate Rinner, Helmut Schaider, Bernadette Liegl-Atzwanger, Katharina Meditz, Julia Preishuber-Pflügl, Sarah Grissenberger, Karl Lohner, Dagmar Zweytick

**Affiliations:** ^1^ Institute of Molecular Biosciences, University of Graz, Graz, Austria; ^2^ BioTechMed, Graz, Austria; ^3^ Biomedical Research, Medical University of Graz, Graz, Austria; ^4^ Cancer Biology Unit, Department of Dermatology, Medical University of Graz, Graz, Austria; ^5^ Dermatology Research Centre, The University of Queensland, School of Medicine, Southern Clinical Division, Woolloongabba, Brisbane, Queensland, Australia; ^6^ Institute of Pathology, Medical University of Graz, Graz, Austria

**Keywords:** human melanoma, antitumor peptide, cancer treatment, phosphatidylserine, mouse xenograft

## Abstract

Di-peptides derived from the human host defense peptide lactoferricin were previously described to specifically interact with the negatively charged lipid phosphatidylserine exposed by cancer cells. In this study one further derivative, namely R-DIM-P-LF11-334 is shown to exhibit even increased cancer toxicity *in vitro* and *in vivo* while non-neoplastic cells are not harmed.

In liposomal model systems composed of phosphatidylserine mimicking cancerous and phosphatidylcholine mimicking non-cancerous membranes the specific interaction with the cancer marker PS was confirmed by specific induction of membrane perturbation and permeabilization in presence of the peptide. *In vitro* studies with cell lines of human malignant melanoma, such as A375, or primary cells of human melanoma metastases to the brain, as MUG Mel1, and non-neoplastic human dermal fibroblasts NHDF revealed high cytotoxic effect of R-DIM-P-LF11-334 on melanoma cells of A375 and MUG Mel1, whereas only minor effect on the dermal fibroblasts NHDF was observed, yielding an about 20-fold killing-specificity for A375 and MUG-Mel1. The LC_50_ values for melanoma A375 and MUG Mel1 were about 10 μM. Analysis of secondary structure of the peptide revealed an increase in the proportion of β-sheets exclusively in presence of the cancer mimic. Stability studies further indicated a potential adequate stability in blood or under stringent conditions. Importantly the cytotoxic effect on cancer cells was also proven *in vivo* in mouse xenografts of human melanoma, where peptide treatment induced strong tumor regression and in average a tumor area reduction of 85% compared to tumors of control mice without peptide treatment.

## INTRODUCTION

New approaches in the treatment of cancer by use of host defense derived peptides, effector molecules of the innate immune system, are of growing interest and have therefore been intensively investigated in the last decade(s) [1–8]. The most important advantage over standard procedures such as surgery, radiation and chemotherapy is the accessibility of and toxicity on dislocated metastases [6, 9], one of the main life threatening forms of cancer. Of further great importance is their specific mechanism, by which these peptides can kill cancer cells. The interaction of the cationic amphipathic peptides with a specific target exposed by cancer cell plasma membranes, the negatively charged lipid phosphatidylserine (PS) [7–12] is in that respect the first important step to exert specific killing efficiency. We were previously able to confirm and extend preceding reports [8, 10, 12, 13] that the normally asymmetric distribution of phospholipids in eukaryotic plasma membranes [14] with a neutral outer leaflet is in the cancer membrane eliminated in favor of a symmetric distribution exposing PS and resulting in a negatively charged outer leaflet [11]. Thus PS exposure has been described for e.g. melanoma and squamous cell carcinoma [12], cancer of ovarian [15], breast and stomach [16, 17] and blood [8]. Our group confirmed these findings for prostate and renal cancer, rhabdomyosarcoma and also for cancer with poor prognosis as malignant melanoma and glioblastoma, cancers to the skin and brain, respectively [11, 18]. Furthermore we could show that PS exposure increased with malignancy thus was highly increased in metastases of melanoma and glioblastoma. Within in this study we could prove that the occurring PS exposure was not due to induced or ongoing apoptosis and was no artefact of cell culturing, since also present in primary cell cultures of cancer [11]. The fact that platelets and erythrocytes also expose PS upon activation seems to cause minimal side effects compared to much higher side effects observed with other applied therapies [7, 19].

Further expedient is the potential treatment of chemo-resistant cancers and the avoidance of side effects due to low toxicity against non-neoplastic cells since derivation of a human host defense peptide. This origins from their specific mode of action to target the plasma membrane [20, 21] where they are supposed to selectively enter cancer cells via PS and reach an inner target, as mitochondria inducing apoptosis [5–7]. Particularly apoptosis is a process that cancer cells learn to circumvent leading to excessive proliferation and resistance. E.g. in metastatic melanoma resistance is reported to be linked to defective apoptosis due to inhibition of expression of a gene encoding Apaf-1, the apoptotic protease activating factor-1 [22]. Thus such cancer types, e.g. malignant melanoma, exhibit only weak sensitivity to chemotherapeutics (10-15%), therefore supplementation by other drugs is a matter of interest.

Though, the specific target PS has been described to be exposed by numerous cancer types, we decided to focus on treatment of a cancer type with poor prognosis and limited therapy options, like malignant melanoma. This cancer shows the highest increase of incidences in the last years. In 1960s the risk to develop malignant melanoma was 1:600, whereas today it is 1:75 to 1:100 [23]. Further it exerts high tendency to metastasize and is mainly chemo-resistant [24, 25]. Thus the long-standing applied treatment with the methylating agent dacarbazine or its oral analogue temozolomide only yielded low response rates of about 15% [26, 27]. New therapies are immunotherapies, as ipilimumab, approved by the FDA in 2011, which blocks the T-cell inhibition of CTLA-4, thereby improving a T-cell mediated immune response against tumor cells. However, it is only able to improve the median survival to 12.7 months [28]. Though, as shown in recent studies it can increase the percentage of 5 years survival of patients treated in combination with dacarbazine to 18% from 8% with solely dacarbazine [29]. Treatment with the also 2011 approved vemurafenib inhibiting the BRAF-serine/threonine-kinase only allows median survival of 13.6 months compared to 9.7 months upon treatment with dacarbazine [30] and in addition causes severe side effects [31, 32]. Further vemurafenib depends on a special mutation in BRAF (V_600_ against E_600_) and is therefore only applicable for 50% of the patients. Vemurafenib has the great advantage of being capable to pass the blood brain barrier and thereby to reach melanoma metastases to the brain, but also the small cationic amphipathic peptides should be capable of that, being however less toxic and acting independently of any mutation.

In our search for novel antitumor therapies, we focused on the human host defense peptide lactoferricin (hLFcin). LFcin is known to exhibit antimicrobial, antiviral, anti-inflammatory and anticancer activities (for a review see [2]). It comprises amino acid residues 1-45 of the N-terminus of human Lactoferrin (hLF). LF11, an 11 amino acid fragment of hLFcin has already been optimized regarding its activity against bacterial [33–35] and cancer cell membranes [6, 9]. So called antitumor active di-peptides were designed with the short LF11 derivatives as one moiety, linked via amino acids like proline to the same moiety (di-peptide) or its reverse sequence (di-retro-peptide) (patent publication number EP2943215 B1 “Lactoferricin derived peptides for use in the treatment of cancer”; application number PCT/EP2014/050330). One, thereby derived antitumor di-retro-peptide is R-DIM-P-LF11-322, which was previously shown to exert activity on PS model systems and antitumor activity on melanoma, glioblastoma and rhabdomyosarcoma without significant toxicity on non-neoplastic cells as melanocytes or dermal fibroblasts [6]. Within this work a correlation of looped β-sheet structure and specific killing of tumor cells by induction of apoptosis was seen [6]. Though toxic interaction of R-DIM-P-LF11-322 with non-neoplastic cells was low, the attempt was made to further decrease its cytotoxicity for the *in vivo* studies. Therefore the peptide was modified by a decrease of its hydrophobicity yielding the peptide R-DIM-P-LF11-334 with two phenylalanines deleted, which is studied in the following *in model*, *in vitro* and especially *in vivo*. Therein we demonstrate that R-DIM-P-LF11-334, does not only exhibit specific activity on PS in model systems and cancer specific toxicity on malignant melanoma *in vitro* but also exhibits strong toxicity on human melanoma *in vivo*.

## RESULTS AND DISCUSSION

### R-DIM-P-LF11-334: the design of an antitumor peptide originated from the human host defense peptide hLFcin

**Figure 1:**
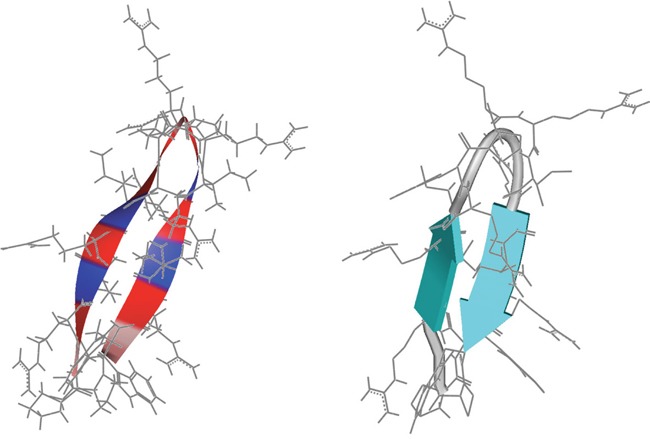
Structure prediction of peptide R-DIM-P-LF11-334 PEP-FOLD secondary structure predictions [40, 41] of peptide R-DIM-P-LF11-334 (PWRIRIRR-P-RRIRIRWP-NH_2_). The amino acids are indicated in grey as backbone. Left: Color code of positions of amino acids in backbone: Arg (red), Ile, Trp (blue) and Pro (pink). Right: The peptide is shown to be predicted to form a β-sheet structure, respectively two β-strands (turquoise) divided by a loop in the middle.

LF11 (amino acid stretch 21-31 of hLFcin, with an exchange of methionine 7 to isoleucine 7) and variants such as LF11-322 were shown to exhibit antibacterial activity correlating with their specific interaction with negatively charged mimics of bacterial membranes as phophatidylglycerol (PG) [33, 34] and cardiolipin (CL) [39]. Since cancer cells also expose a negatively charged lipid in form of phosphatidylserine (PS) [10–12], antibacterial short peptides (8-12 amino acids) were also tested for their antitumor activity. However, the short peptides were not antitumor-active *in vitro*, probably due to lack of formation of a stable secondary structure [6, 9]. Thus so called di-peptides were designed with the short LF11-derivatives as one moiety, linked via amino acids as proline to the same moiety (di-peptide) or its reverse sequence (di-retro-peptide) [6]. One of thereby derived antitumor di-retro-peptides, R-DIM-P-LF11-322, was previously shown to exert activity on PS model systems and antitumor activity on melanoma and other cancers without significantly harming non-neoplastic cells [6]. Within the study a correlation of its looped β-sheet structure and the specific killing of tumor cells by induction of apoptosis was demonstrated [6]. Though toxic interaction of R-DIM-P-LF11-322 with non-neoplastic cells was low, the attempt was now made to further decrease the cytotoxicity by a decrease of its hydrophobicity, enhancing the potential of peptides for therapeutic applications. Therefore, the two phenylalanines (F) of R-DIM-P-LF11-322 located close to the C- and N-termini were removed resulting in the design of R-DIM-P-LF11-334 (moiety LF11-334; primary sequences see Table 1). Like R-DIM-P-LF11-322 also R-DIM-P-LF11-334 forms a stable β-sheet structure as predicted with the online program PEP-fold (http://bioserv.rpbs.univ-paris-diderot.fr/PEP-FOLD/) [40–42] (see Figure 1). In the following the newly designed peptide will be characterized in its interaction with cancer and non-cancer model systems, melanoma cells, their metastases and normal dermal fibroblasts *in vitro* and finally human melanoma in mouse xenografts *in vivo*.

**Table 1:** Overview of peptide sequences, net charge and positive charges of hLFcin derivatives

Peptide	Sequence	length [aa] / net charge / No of R or K
LF11-322	**PFWRIRIRR**-NH_2_	9 / +5 / 4R
R-DIM-P-LF11-322	**PFWRIRIRR**-P-RRIRIRWFP-NH_2_	19 / +9 / 8R
**LF11-334**	**PWRIRIRR**-NH_2_	8 / +5 / 4R
**R-DIM-P-LF11-334**	**PWRIRIRR**-P-RRIRIRWP-NH_2_	17 / +9 / 8R

Abbreviations: Aa, amino acid; No, number; R, arginine; K, leucine

### R-DIM-P-LF11-334: specific activity on cancer mimic PS *in model* systems

**Figure 2:**
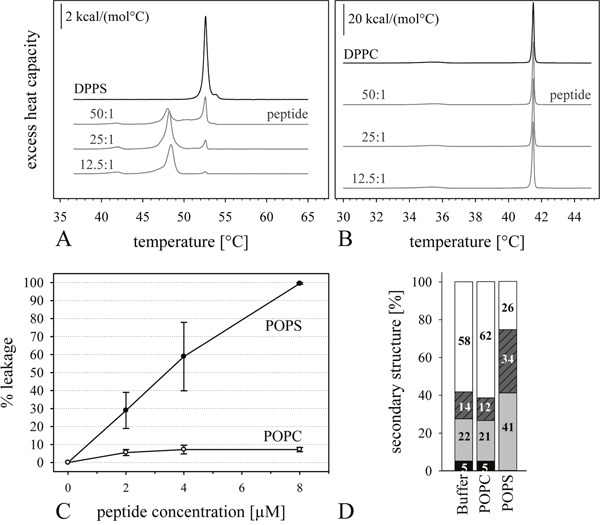
Influence of peptide R-DIM-P-LF11-334 on model systems of cancer and non-cancer cells (**A** and **B**): **DSC thermograms** of cancer mimic DPPS **(A)** or non-cancer mimic DPPC **(B)** in the absence (black) and presence of the peptide R-DIM-P-LF11-334 (gray) (50:1, 25:1 and 12.5:1 lipid-to-peptide molar ratios). For clarity, the DSC curves were displayed on the ordinate by an arbitrary increment. The peptide shows increasing activity with increasing peptide ratios only on the cancer model PS. **(C)**: **ANTS/DPX leakage** of LUVs composed of cancer mimics POPS (−●-) or non-cancer mimic POPC (−○-) as a function of concentration of R-DIM-P-LF11-334. Concentration of LUVs was 50 μM and temperature was kept at 37°C during measurements. The peptide only shows strong interaction with the cancer mimic system PS. **(D)**: **CD-spectra**-Secondary structures of R-DIM-P-LF11-334 in HEPES buffer or presence of LUVs of cancer mimic POPS and non-cancer mimic POPC at lipid-to-peptide ratio of 100:1 calculated from CD spectra using Dichroweb, Contin-LL (Provencher & Glockner Method) Convolution Program [37, 38]. The α-helical content is shown in black; β-turns are shown in light grey; turns are shown in dashed dark grey; random coil structures are shown in white. The peptide R-DIM-P-LF11-334 changes its secondary structure only in the presence of the cancer mimic POPS. No change of secondary structures in the presence of the healthy mimic POPC was observed as compared to buffer.

Analysis of the peptide interaction with simple model systems composed of PS mimicking cancer cells and model systems composed of PC mimicking non-neoplastic cells are first steps in order to gain information on the membrane specificity of our antitumor peptide. As can be deduced from differential scanning calorimetry (DSC) experiments (Figure 2 and Table 2), R-DIM-P-LF11-334 shows strong and specific effect on the thermotropic phase behavior of the cancer mimic DPPS (Figure 2A), whereas no effect is exhibited on the non-cancer mimic DPPC (Figure 2B). Severe membrane perturbation was observed for the cancer mimic DPPS in presence of the peptide. The transition temperature (T_m_) of the lipid was shifted to lower temperatures by several degrees. The transitions were mainly split in 2 or more peaks, due to peptide affected lipid domains, where the lower temperature domain is presumably more highly enriched in peptide. The cooperativity of this domain was decreased, indicated by the increase of the half-width (ΔT_1/2_). The proportion of unaffected domains with a transition temperature near that of pure DPPS (52.6°C) was decreasing with increasing amounts of peptide (50:1, 25:1 to 12.5:1 lipid to peptide, molar ratios). Concomitantly, the fraction of peptide affected domains increased with a decrease in transition temperature to about 48°C (see Table 2) indicating a concentration dependent peptide effect on the cancer mimic. Further the decrease of the total phase transition enthalpy (ΔH_cal_) indicated severe membrane destabilization of the cancer mimic by the peptide R-DIM-P-LF11-334. In contrast, the peptide had no effect at all on the “healthy” mimic DPPC at any lipid-to-peptide molar ratio studied (see Figure 2B).

**Table 2:** Thermodynamic parameters of DDPS (cancer cell mimic) in the absence and presence of R-DIM-P-LF11-334 at a lipid to peptide molar ratio of 50:1, 25:1 and 12.5:1

A	Δ*H*_m_[kcal/mole]	T_m_[°C]	T_1/2_[°C]
**DPPS**	11.5	52.6	0.50
**+ R-DIM-P-LF11-334 50:1**	5.5 / 4.8	48.0 / 52.6	0.90 / 0.40
**+ R-DIM-P-LF11-334 25:1**	8.7 / 1.0	48.2 / 52.6	0.80 / 0.40
**+ R-DIM-P-LF11-33412.5:1**	7.5 / 0.2	48.4 / 52.6	0.90 / 0.40

Data were analyzed from thermotropic phase behaviors illustrated in Figure 2A using MicroCal Origin software (VP-DSC version).

Consistently as demonstrated in Figure 2C ANTS/DPX leakage is only induced on liposomes composed of the cancer mimic POPS, whereas POPC liposomes are hardly permeabilized by the peptide. At 8 μM peptide concentration 100% of the POPS liposomes show leakage compared to less than 10% of the POPC liposomes.

A specific interaction of R-DIM-P-LF11-334 with the cancer mimic PS was also confirmed by CD-spectroscopy-studies (Figure 2D). In solution and in absence of a (cancer) target membrane as e.g. the healthy mimic POPC the peptide was mainly unstructured (∼60%) or partially adopted a β-sheet structure. Similarly, as was also shown for the cancer specific peptide R-DIM-P-LF11-322, R-DIM-P-LF11-334 only changes its structure in the presence of the cancer mimic POPS by increasing the proportion of β-turns (20% to 40%) by in return decreasing the proportion of non-ordered structures. This change of structure detected only in the presence of the target membrane seems to be one of the driving forces for a specific interaction with tumor cells exposing PS. As shown for the peptide DIM-LF11-318, which forms the same secondary structure (α-helix) in the presence of both, the cancer and the non-cancer mimic, a non-specific structure formation leads to toxicity on neoplastic and non-neoplastic cells [6].

### R-DIM-P-LF11-334: stability in presence of blood components and extreme pH conditions

**Figure 3:**
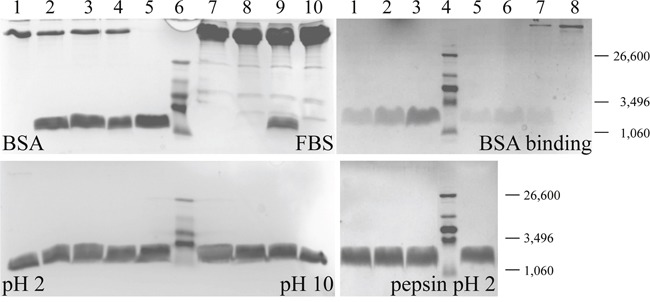
Stability and binding studies on peptide R-DIM-P-LF11-334 Left: SDS-polyacrylamide gel electrophoresis of different peptide stability studies as obtained in the presence of BSA (upper left), FBS (upper right), in buffer pH 2 (left) and pH 10 (right). Lane 1 or 10 in top: Respective controls (BSA, FBS) without peptide. Peptide samples were incubated in the presence of the respective components at 37°C for 1 day (lane 2 and 9), 6 days (lane 3 and 8) and 7 days (lane 4 and 7). Lane 5, as well as lane 1 and 10 in bottom gel: Solely peptide R-DIM-P-LF11-334. Molecular weights can be derived from lane 6 loaded with ultralow molecular weight standard, fragment sizes are given at the right. Right: Top: SDS-polyacrylamide gel electrophoresis of peptide binding study as obtained in the presence of BSA. Lane 1-3: 0.25 μg, 0.5 μg and 1 μg solely peptide R-DIM-P-LF11-334. Peptide samples were incubated in the presence of BSA at 37°C for 7 days. Free (unbound) peptide (0,165 μg*) (lane 5), control free peptide (0.64 μg*) (lane 6) and BSA-bound peptide (lane 7) are shown. Lane 8: BSA without peptide. *Determined by measurement of UV-absorbance of tryptophan at 280 nm. Bottom: Peptide samples were incubated in the presence of pepsin at 37°C for 1 day (lane 1), 2 days (lane 2) and 7 days (lane 3). Lane 5 shows solely peptide R-DIM-P-LF11-334. Lane 4 in top and bottom gel: Ultralow molecular weight standard.

For an application *in vitro* and *in vivo*, stability and availability in presence of proteins and other components in blood or stringent conditions in stomach can be crucial factors. On the one hand, the effective concentration of the peptides at the tumor cells could be decreased by proteolytic or chemical degradation before completion of action of the peptide; on the other hand however, the half-life of the applied drug during transport in blood could even be enhanced by binding to serum proteins. A possible degradation was measured in the following (Figure 3). One or more components in fetal bovine serum (FBS), other than BSA, were seemingly able to reduce the amount of peptide, however only after more than 1 day of incubation. After 2 days about 1/3 of the peptide was still left (data not shown), after 6 days no peptide was detectable anymore. The peptide might be degraded by components in the serum. However for a period of at least 24 hours the peptide can exert its complete activity in the presence of FBS, which seems efficient (see also next section *in vitro* activity).

BSA itself had no degrading effect on the peptide, which would not have been expected, though was tested since it's a main component of FBS used in the cytotoxicity studies (next section). BSA is sometimes functioning as a so called “molecular taxi” in the organism, transporting fatty acids or various drugs through the blood system. This would be even beneficial for an increase of the peptide half-life. Respective binding studies however necessitate native conditions during gel electrophoresis or extra pre-separation of unbound and BSA bound peptide. The latter was performed and revealed a binding of approximately 75% of the peptide to BSA after 7 days of incubation (Figure 3, right side; lane 5, free peptide and lane 7, BSA bound peptide). Anyhow, the presence of BSA and FBS and potential binding, as well as starting degradation, after 24 hours did not seem to hinder the high cytotoxic effect of the peptide on cancer cells during treatment being complete within this time (next section *in vitro* activity). This was confirmed by toxicity studies in presence of decreasing amounts of serum (10%, 2% and 0%), which had no significant effect on peptide activity on melanoma cells *in vitro* after 8 hours. Only the killing velocity was increased in the absence of serum (data not shown).

Also different pH, as highly acidic conditions at pH 2, mimicking conditions in the stomach, or highly basic conditions at pH 10 did not have any impact on the intactness of the peptide thus induce any degradation (Figure 3). For oral application, additional stability studies in presence of pepsin at pH 2 were performed and revealed no visible cleavage. However, cutting at respective pepsin binding sites proposed by the program “Expasy Peptide Cutter” (http://web.expasy.org/peptide_cutter/) at position 1 and 16 of R-DIM-P-LF11-334 would only cleave off the first and the last amino acids and be hardly observable on the SDS page. Respective studies of *in vitro* toxicity in presence of pepsin are therefore an ongoing study. Besides, the higher damage by cleavage in presence of trypsin or chymotrypsin in intestine will anyhow demand for further protection of the peptide by e.g., encapsulation in coated nanoparticles for potential oral application.

Summarized, the preliminary stability and binding experiments with R-DIM-P-LF11-334 indicate potential adequate stability in blood or under stringent conditions, which might be important upon intravenous application.

### R-DIM-P-LF11-334: specific activity on malignant melanoma cells *in vitro*

**Figure 4:**
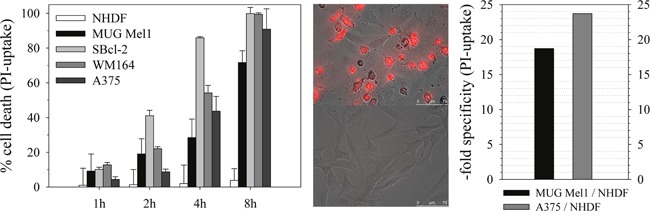
Cytotoxicity and specificity of peptide R-DIM-P-LF11-334 Left: Cytotoxicity on cell lines of primary cells of melanoma metastases MUG Mel1, non-tumorigenic melanoma SBcl-2, melanoma metastases WM164, melanoma A375 and normal human dermal fibroblasts NHDF from 1-8 hours of incubation with 20 μM of peptide. High cell death of the melanoma cells and metastases is induced after 8 hours of peptide incubation, whereas the non-neoplastic cell line NHDF is nearly non-affected. Middle: Overlay of fluorescence micrographs and bright field of melanoma metastases MUG Mel1 (top) and dermal fibroblasts NHDF (bottom) are shown after 4 hours of peptide incubation at a concentration of 20 μM. Red dye indicates PI-uptake upon peptide induced membrane disintegration occurring specifically in melanoma cells. Right: X-fold specificity of peptide R-DIM-P-LF11-334 at 20 μM peptide concentration after 8h of incubation displayed as PI-uptake ratio of A375 vs. NHDF and MUG Mel1 vs. NHDF. The peptide kills melanoma cells with high specificity.

**Figure 5:**
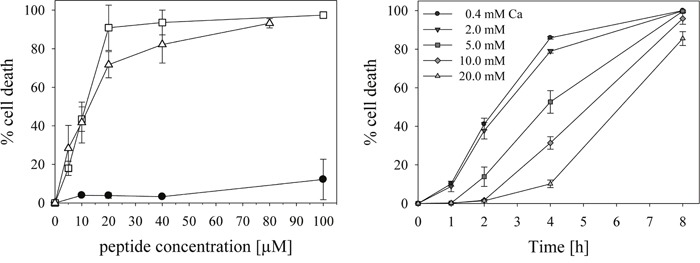
Cytotoxicity of peptide R-DIM-P-LF11-334 Left: The lines show cell death induced by increasing peptide concentrations determined by % PI-uptake of cell lines A375 (−□-), MUG Mel1 (−Δ-) and NHDF (−●-) after 8h of incubation. For LC_50_ values see Table 3. Right: Cell death determined by PI-uptake of melanoma of primary lesions SBcl-2 in the presence of 20 μM peptide and increasing amounts of Ca^2+^ (0.4-20 mM). The activity of the peptide R-DIM-P-LF11-334 is affected by increasing concentrations of Ca^2+^, indicating competing interaction with negative charges on the surface of cancer cells for at least 4 hours.

Next, the toxicity of R-DIM-P-LF11-334 on malignant melanoma cell line A375, primary lesions of melanoma SBcl-2, melanoma metastases WM164 and the primary cells of melanoma brain metastases MUG Mel1 was tested. To exclude possible peptide toxicity on non-neoplastic cells also effects on normal human dermal fibroblasts NHDF were analyzed (Figures 4 and 5 and Table 3).

**Table 3:** Comparison of LC_50_ values [μM] of treated A375, MUG Mel1 and NHDF determined through PI-uptake (8 h) and MTS cell viability assay (24 h)

PeptideR-DIM-P-LF11-334	MelanomaA375	MelanomaMUG Mel1	Dermal FibroblastsNHDF
LC_50 PI_ [μM]LC_50 MTS_ [μM]	10.3±0.626.7±1.9	11.3±0.813.6±0.2	>100> 90

Data for PI measurements from at least five and for MTS from at least three experiments are presented as mean ± SD.

As could be seen by PI-uptake of the different melanoma cell lines significant cell death was induced by the peptide, but only after 4 −8 hours of peptide incubation (Figure 4, left, middle). This slow induction of cell death is in accordance with the slow killing velocity exhibited by the related peptide R-DIM-P-LF11-322 with an additional F on second and penultimate position, where the slow killing was correlated with induction of apoptosis [6]. The non-neoplastic control cells NHDF were only minor affected by the peptide R-DIM-P-LF11-334 (Figure 4, left). Hence a high killing specificity of about 20-fold for the melanoma (A375) and even their metastases (MUG Mel1) over the non-neoplastic cells (NHDF) was achieved for a peptide concentration of 20 μM after 8 hours of incubation (Figure 4, right).

Upon treatment with different peptide concentrations the LC_50_ values, gained by analysis of PI-uptake (cell death) for the different cell types, were about 10 μM for the melanoma cell lines and higher than 100 μM for the non-neoplastic cell line. This relates to a more than 10-fold cancer specificity (Figure 5, left and Table 3). LC_50_ values of the peptide gained by analysis of remaining cell viability (MTS) showed that the peptide is still more than 3-6-fold specific for cancer cells (Table 3). This specificity for cancer cells is increased for R-DIM-P-LF11-334 compared to the related peptide R-DIM-P-LF11-322 [6] by a factor of 1.5 and 1.2 as studied by induced cell death (PI-uptake) or remaining cell viability (MTS), respectively. As reported previously for the cancer specific peptide R-DIM-P-LF11-322, also the first interaction of R-DIM-P-LF11-334 with the cancer membrane seems to be mainly driven by electrostatic attraction, since the toxicity of the peptide on melanoma cell line SBcl-2 is significantly affected by the presence of Ca^2+^ ions at the beginning. Thus, up to 4 hours the PI-uptake of SBcl-2 in the presence of 20 μM R-DIM-P-LF11-334 is reduced from 86% at normal level of Ca^2+^ (0.4 mM) to 10% at the highest concentration of Ca^2+^ tested (20 mM) indicating a strong competition of the peptide and Ca^2+^ for a negatively charged target on the cancer membrane at the beginning. This supports the mechanistic model that the internalization of the cationic peptides into the cancer cells is driven by electrostatic interaction with negatively charged membrane components such as PS. Similar to R-DIM-P-LF11-322 after 8 hours Ca^2+^ does no longer affect peptide activity, allowing a high killing efficiency independent on the presence of Ca^2+^, probably due to the fact that the peptide can either displace the Ca^2+^ or Ca^2+^ has already been internalized with time. This is also in agreement with the proposed mechanism that after specific entrance into the cancer cell inner targets as mitochondria are attacked and cell death by apoptosis is induced [6]. Again negatively charged lipids, like PS or cardiolipin can be targets on these organelles. As also shown in the previous work activity of the non-selective peptide DIM-LF11-318 was not affected by Ca^2+^ indicating additional hydrophobic interactions occurring and thus electrostatic interactions at the beginning to be a main driving force for a specific entrance of peptides into the cancer cell, as proposed for R-DIM-P-LF11-322 [6] and now R-DIM-P-LF11-334. This is further in good correlation with the specific interaction of R-DIM-P-LF11-334 with PS model systems described.

### R-DIM-P-LF11-334 induces tumor regression of human malignant melanoma in mouse xenografts *in vivo*

**Figure 6:**
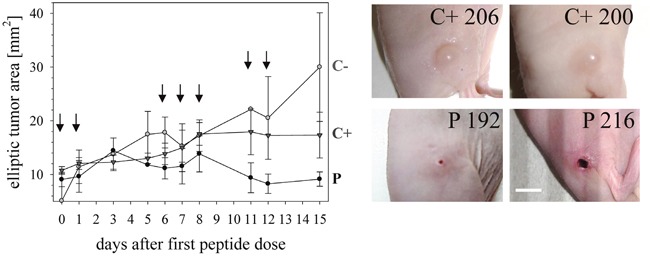
Tumor growth in melanoma xenografts treated or non-treated with peptide R-DIM-P-LF11-334 20 FOXN1 mice (Charles River Laboratories, Sulzfeld, Germany) were subcutaneously xenotransplanted with A375 cells in the right flank. The tumors were grown to an approximate maximal length and width of 3 mm before peptide or control treatment was started (day 0). Left: 10 mice were treated with peptide (P; -●-) dissolved in buffer PBS, 5 mice were treated with only PBS (C+; -▼-), 5 mice were kept untreated (C-; -●-). Treatment occurred as indicated by arrows at days 0, 1, 6, 7, 8, 11 and 12 in 7 single doses by injection into the tumor. The total dose per mouse was 1.67 mg. The tumor sizes were measured with manual calipers during the treatment time span. Thereby the width (2*a_ell_) and length (2*b_ell_) of the tumors were determined and the tumor areas were calculated as ellipses (A_ell_=a*b*π) illustrated as medium values for P, C+ and C- with standard deviations per time point. Right: Pictures of human subcutaneously grown melanoma of two representative control mice that were treated with buffer PBS (C+ 206 and C+ 200) and two mice treated with peptide (P 192 and P 216). The tumor areas and sizes are significantly reduced by treatment with peptide R-DIM-P-LF11-334. Bar represents 0.5 cm.

**Figure 7:**
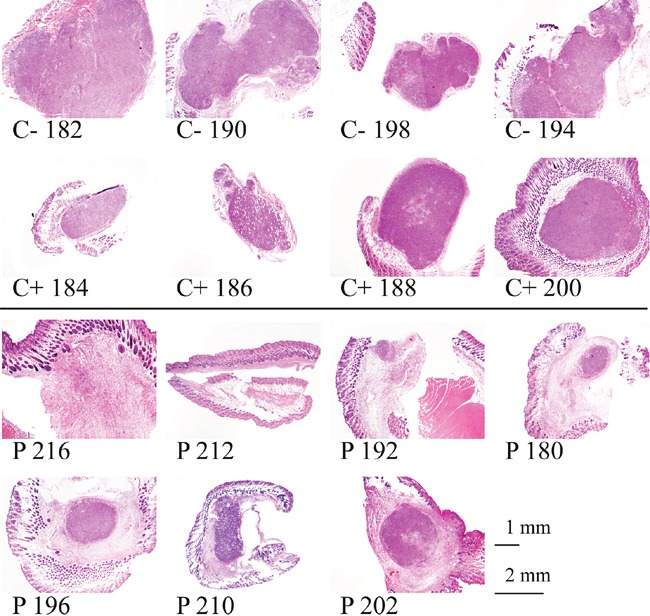
Histological staining of tumors of melanoma xenografts treated or non-treated with peptide R-DIM-P-LF11-334 At day 15 of peptide treatment the mice were sacrificed and the tumors were isolated and examined after paraffin embedment. Sections were stained with hematoxylin and eosin (C- no treatment, C+ intratumoral injection of buffer PBS, P treatment by intratumoral peptide injection). Sections of peptide treated tumors (P) show tumor regression and shrinkage with highly reduced tumor areas (dark violet), whereas in control tumors (C+ and C-) the areas are larger and cover the whole area of the biopsied sections.

**Figure 8:**
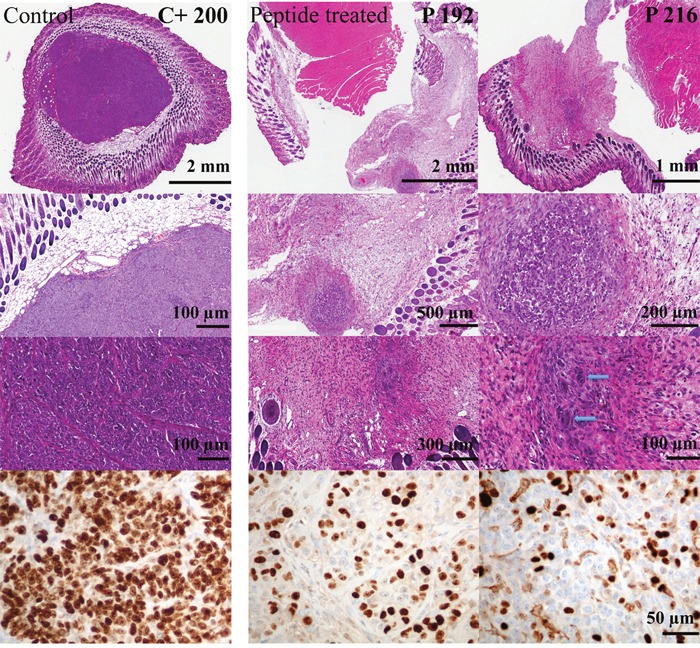
Cell proliferation of tumors of melanoma xenografts treated or non-treated with peptide R-DIM-P-LF11-334 Different magnifications of tumor sections stained with hematoxylin and eosin are shown (Top, 3 rows). The dark violet areas indicate dense growth of tumor cells in the entire area of the biopsy of the control mice only treated with buffer PBS (left, C+ 200). The peptide treated tumor sections show massive fibrosis and only remnants of tumor tissue. Blue arrows indicate only single residual tumor cells in the center. Bottom: Staining of Ki-67 reveals significant reduction of highly proliferative tumor cells upon peptide treatment (middle and right, P 192 and P 216) compared to excessive proliferation in control cells without peptide treatment (left, C+ 200); scale bar at the right side is applicable for the bottom row; C+ intratumoral injection of PBS, P treatment by intratumoral peptide injection.

Because of its highly selective activity of R-DIM-P-LF11-334 for cancer cells the peptide was further evaluated *in vivo* studies. Therein 20 FOXN1 mice were xenotransplanted with human melanoma A375 cells subcutaneously in the right flank. Tumor growth was permitted to reach an elliptic area up to approximately 4-10 mm^2^. Peptide (P) or control (C) treatment (buffer PBS C+, none C-) was then started simultaneously at days 0, 1, 6, 7, 8, 11 and 12 in 7 single doses by injection into the tumor. The tumor sizes were measured during the treatment time span (Figure 6). At day 15 the mice were sacrificed and the tumors were isolated and examined together with the organs upon paraffin embedment and staining of sections with hematoxylin and eosin (Figures 7 and 8). Furthermore in some cases Ki67 staining was performed for determination of excessive cell proliferation (Figure 8).

External measurements of the tumors (Figure 6) indicated that the peptide treatment P stopped tumor growth, whereas tumors continued growing in control mice C+ (PBS) or even more in control mice C- (none). Summarized after treatment the tumor sizes were significantly reduced in mice treated with peptide compared to control mice.

At day 1 and day 3 after the application of the first and second intratumoral peptide treatment in some cases a wound developed in the area of the tumor (Figure 6, P 216). However, fast healing and formation of a scab occurred. In these cases an external tumor measurement was difficult to perform. Therefore, the initial dose of 0.33 mg was reduced to 0.20 mg for the following treatments applied with a 4 days pause to avoid formation of larger wounds or stress.

**Figure 9:**
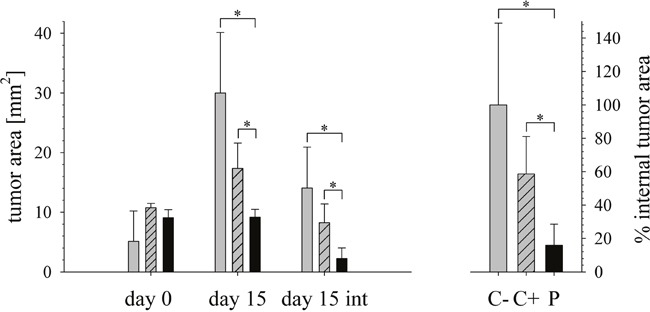
Effect of peptide treatment on tumor size measured externally or internally Left: Tumor growth of mouse xenografts of A375 is shown measured externally at day 0 (start of treatment) and externally and internally (int) at day 15 (end of treatment). C- no treatment (grey bars; 5 mice), C+ intratumoral injection of PBS (dashed grey bars; 5 mice), P treatment by intratumorale peptide injection (black bars; 9 mice). Right: Peptide treatment caused stop of tumor growth and a significant reduction of the tumor size as shown by histological staining. The accomplished decrease of internal tumor size upon peptide treatment was about 85% compared to tumor size in mice without treatment. Bars: C- no treatment (grey bars), C+ PBS (dashed grey bars), P treatment (black bars). *Tumor size decrease in peptide treated mice (P) at day 15 measured externally and internally was statistically significant compared to control tumor size C- or C+, as analyzed by the student's t test (*p < 0.001). All values are expressed as a mean ± SEM.

After day 15 when the tumors were biopsied and stained with hematoxylin and eosin (Figure 7 and 8) the internal tumor sizes could be elucidated by determination of stained areas of the sections (Figure 9).

All evaluated samples showed a predominantly epithelioid morphology with large nuclei and prominent nucleoli (Figure 7). The tumor was located in the dermis and partly in between the skeletal muscle. In absence of treatment (C-) however they exhibited irregular contours, whereas upon injection of buffer (C+) or peptide (P) the contours were more regular and circular. Comparing the tumors of control mice C- without treatment and C+ with intratumoral injection of PBS, it was striking that already the injection of PBS into the tumor had an effect on tumor size and tumor morphology. The externally measured size of the tumor got decreased in these cases (Figure 6; C+). The C+ tumors also got stiffer, as observed during treatment. However as shown by histological staining the tumor area covered the whole area of the biopsied tumor when no peptide was applied. Moreover upon peptide treatment the tumor size did not only decrease when measured from the outside, but was even decreased more dramatically when measured inside according to the stained area (Figure 6, 7, 8 and 9). Further in all cases of peptide treatment a significant and massive fibrosis occurred. This fibrosis mainly surrounded a massively shrunk tumor tissue, which was in some tumors only composed of up to a few tumor cells (P 216, blue arrows Figure 8) or a small (< 1 mm diameter) focal tumor node of epithelioid tumor cells (P 192) (Figure 7 and 8). As also observed during treatment the tumor was softer when treated with peptide than with PBS probably due to the regrowth of connective tissue in the latter. In control tumors without PBS, fibrosis was minimal, in control tumors with PBS only slight focal fibrosis occurred. Thus the tumor sections consisted mainly of tumor tissue. Also all peptide treated tumors showed significant regressive changes, which may have resulted in disappearance of malignant cells, as well as also partially focale myxoide loosening (P 210). As elaborated all peptide treated tumors showed significant decrease of the tumor area. Staining of Ki-67 of the tumor of the control mouse C+ 200 and staining of tumors of peptide treated mice P 192 and P 216 indicated that malignant cell proliferation was massively decreased in presence of peptide (Figure 8). Thus high proliferative activity was only observed in the control samples indicating ongoing tumor growth (up to 98%) whereas in the peptide treated samples the Ki-67 staining was low due to reduced or inhibited tumor growth. Necrosis was never present. A reduction of tumor size induced by other antitumor agents, the pleurocidin family peptides NRC-03 and NRC-07 was reported by Hilchie et al., [43] in breast cancer xenografts. Contrary, upon treatment with the pleurocidin-family peptides increasing necrosis in the tumors was reported. Interestingly in their study the tumors showed necrotic cells surrounded by tumor tissue reaching the outside, which was however loosened upon treatment [43]. A further antitumor peptide, LTX-315, was reported to induce necrosis in B16 melanoma mice [44]. Similarly intratumoral LTX-302 injection resulted in tumor necrosis and infiltration of inflammatory cells followed by complete regression of the tumors in syngeneic mice carrying lymphoma [45]. LTX-315 and LTX-302 were derived by structural optimization of bovine lactoferricin, though containing the non-coded residue β-diphenylalanine [44, 45]. *In vivo* studies in 2006 with 2 mg of original bovine lactoferricin injected intratumoral into neuroblastoma xenografts only caused tumor growth inhibition, not tumor size decrease [46].

In our study tumor growth of mouse xenografts of A375 was in average inhibited in peptide treated mice as determined by external measurement at day 0 (start of treatment) and day 15 (end of treatment), whereas in control mice without buffer C- tumors grew to sizes 6-fold and in control mice with PBS C+ to 1.8-fold of those at day 0 (Figure 9). Analysis of internal tumor sizes determined by histological staining however yielded a strong reduction of the tumor size upon peptide treatment of in average 85% compared to non-treated tumors of control mice. The peptide treated tumor area was in average only 2.2 mm^2^, whereas the average area of untreated control tumors was 14 mm^2^ and of PBS treated was 8.2 mm^2^. Statistical analysis of the data yielding a p-value smaller than 0.001 proved significance of the differences observed in tumor sizes upon peptide treatment compared to control mice. Thus a strong effect of the peptide *in vivo* by inhibition of tumor growth and tumor regression was shown.

Complete blood count of control mice treated with PBS and mice treated with peptide were studied and were comparable (Supplementary Table 1). In comparison with normal blood values, though not completely applicable for immunocompromised mice it is however striking, that without peptide treatment the mouse xenografts showed elevated levels for leukocytes, mean corpuscular volume (MCV), hematocrit and thrombocytes, whereas the levels of the peptide treated mice were in range with only slightly increased levels of lymphocytes and increased levels of thrombocytes.

To test further potential side effects of the peptide *in vivo*, 3 mice were injected subcutaneously into the flank with 9 doses of peptide to a total amount of 0.68 mg per mouse over 3 weeks. Then the skin at injection site, liver, kidney, spleen, and weight were checked for abnormal changes (Supplementary Figure 1, liver and kidney). No inflammation occurred, all organs were unsuspicious and weight gain was normal.

## MATERIALS AND METHODS

### Materials

The peptide R-DIM-P-LF11-334 (PWRIRIR RPRRIRIRWP-NH_2_, M= 2382.4 g/mol) was purchased from PolyPeptide. (San Diego, CA, USA). The purity was >96% as determined by RP-HPLC. The peptide was dissolved in acetic acid (0.1%, v/v) at concentrations of 3 mg/ml for *in vitro* experiments and in phosphate buffered saline (PBS, 20 mM NaPi, 130 mM NaCl, PH7.4) buffer at concentrations of 3.3 mg/ml for *in vivo* experiments. Peptide solutions were stored at 4°C and concentrations were determined by measurement of UV-absorbance of tryptophan at 280 nm.

1,2-dipalmitoyl-sn-glycero-3-phosphocholine (DPPC), 1-palmitoyl-2-oleoyl-sn-glycero-3-phosphocholine (POPC), 1,2-dipalmitoyl-sn-glycero-3-phospho-L-serine (Na-salt) (DPPS) and 1-palmitoyl-2-oleoyl-sn-glycero-3-phospho-L-serine (Na-salt) (POPS) were purchased from Avanti Polar Lipids, Inc. (USA), and used without further purification. Stock solutions of DPPC and POPC were prepared in CHCl_3_/CH_3_OH (2:1, v/v), stock solutions of DPPS and POPS were prepared in CHCl3/CH3OH (9:1, v/v) and stored at −18°C.

ANTS (8-aminonaphthalene-1,3,6-trisulfonic acid, disodium salt) and DPX (p-xylene-bis-pyridinium bromide) used for permeability studies were purchased from Thermo Fischer Scientific (Molecular Probes, USA).

### Cell culture

The human malignant melanoma cell line A375 was purchased from ATCC (American Type Culture Collection, Manassas, Virginia, US) and cultured in Dulbecco's Modified Eagle Medium with GlutaMAX™ (DMEM, Gibco^®^, Thermo Fisher Scientific, USA) with addition of 10% FBS (Gibco^®^). Melanoma cell lines from primary (SBcl-2) and metastatic (WM164) lesions (kindly provided by Dr. Meenhard Herlyn, the Wistar Institute, Philadelphia, PA) and newly established primary cells MUG-Mel1 (manuscript Rinner et al., in preparation) were maintained in RPMI 1640 medium with GlutaMAX™ (Gibco^®^, Thermo Fisher Scientific, USA) supplemented with 2% FBS (fetal bovine serum; Gibco^®^). Normal human dermal fibroblasts NHDF, used as healthy control cells, purchased from PromoCell GmbH were cultured in fibroblast growth medium 2 (PromoCell GmbH). All cells were kept in a 5% CO_2_ atmosphere at 37°C. At 90% confluence cells were passaged with accutase (Gibco^®^, Thermo Fisher Scientific, USA). All cell cultures were periodically checked for mycoplasma. Low passages were used for *in vitro* studies with MUG-Mel1 (passage 3-7) and NHDF (passage 2-7).

### Differential scanning calorimetry (DSC)

For preparation of liposomes 1 mg of respective phospholipid stock solution was dried under a stream of nitrogen and stored in vacuum overnight to completely remove organic solvents. The dry lipid film was then dispersed in phosphate buffered saline (PBS, 20 mM NaPi, 130 mM NaCl, pH 7.4) and hydrated at a temperature well above the gel to fluid phase transition of the respective phospholipid under intermittent vigorous vortex-mixing. The lipid concentration was 0.1 weight% for calorimetric experiments. Hydration was carried out in presence or absence of peptide at a lipid-to-peptide molar ratio of 50:1, 25:1 and 12.5:1 using a protocol described for 1,2-dipamitoyl-sn-glycero-3-phospho-L-serine (DPPS) [9]. Briefly hydration of DPPS films was performed at 65°C for two hours with vortexing for 1 minute every 15 minutes. DPPC liposomes were hydrated at 50°C with vortexing every 15 minutes for two hours. The fully hydrated samples were stored for at least 1 hour at room temperature until measurement. DSC experiments were performed with a differential scanning calorimeter (VP-DSC) from MicroCal, Inc. (Northhampton, MA, USA). Heating scans were performed at a scan rate of 30°C/h (pre-scan thermostating 30 minutes) with a final temperature of approximately 10°C above the main transition temperature (Tm) and cooling scans at the same scan rate (pre-scan thermostating 1 minute) with a final temperature approximately 20°C below Tm. The heating/cooling cycle was performed three times. Enthalpies were calculated by integration of the peak areas after normalization to phospholipid concentration and baseline adjustment using the MicroCal Origin software (VP-DSC version).

**ANTS/DPX leakage**—Leakage of aqueous contents from liposomes composed of POPS (1-palmitoyl-2-oleoyl-*sn*-glycero-3-phospho-L-serine) or POPC (1-palmitoyl-2-oleoyl-*sn*-glycero-3-phosphocholine) in the presence of increasing amounts of peptide (2 μM, 4 μM and 8 μM) was determined using the 8-aminonaphthalene-1,3,6-trisulfonic acid / p-xylene-bis-pyridinium bromide (ANTS/DPX) assay. Lipid films were prepared, hydrated, extruded, measured and analyzed, as described previously [9, 36].

**Circular dichroism (CD)**—Measurements were performed on a Jasco J 715 Spectropolarimeter (Jasco, Gross-Umstadt, Germany) at room temperature using quartz cuvettes with an optical path length of 0.02 cm. The CD spectra were measured between 260 nm and 180 nm with a 0.2 nm step resolution. To improve accuracy 3 scans were averaged. Peptide was dissolved in 10 mM Hepes (pH 7.4) to a final concentration of 200 μM. Spectra were measured in the absence and presence of LUVs of 20 mM POPS and 20 mM POPC (Avanti Polar Lipids, Alabaster, USA) mimicking cancer and healthy mammalian membranes, respectively. The respective lipid to peptide molar ratio was 100:1. Background signals were abstracted after measurements [9]. Percentage secondary structure calculations were done using Dichroweb, Contin-LL (Provencher & Glockner Method) Convolution Program using reference set 7 [37, 38].

### Stability and binding tests

Peptide stability was tested in presence of bovine serum albumin (BSA, Sigma-Aldrich, Deisenhofen, Germany), fetal bovine serum (FBS, Gibco^®^), buffer pH 2 (30 mM citric acid/HCl, 60 mM NaCl), pH 10 (10 mM Tris/NaOH), and pepsin from porcine gastric mucosa (Sigma-Aldrich, Deisenhofen, Germany) in buffer pH 2. Therefore peptide samples were incubated in presence of 1:1 weight ratios (10:1 in presence of pepsin) with the mentioned components or in presence of buffer (pH 2 or 10) for 1 day, 6 days and 7 days at 37°C. After the given time points aliquots were taken and diluted with electrophoresis loading buffer (2.5% SDS) heated for 5 minutes at 95°C and analyzed by SDS-gel electrophoreses (BioRad, Mini-PROTEAN Tetra System). As standard the Ultra-low Range Molecular Weight Marker M3546 (Sigma-Aldrich, Deisenhofen, Germany) was used. For analysis of binding to BSA after 7 days respective samples were taken and loaded on Amicon Ultra-0.5 ml Centrifugal Filters (Ultracel-10K) (Merck Millipore, Tullagreen, IRL). After centrifugation (14.000g, 30 minutes, 4°C) the filtrate was analyzed for amount of non-BSA-bound peptide by measurement of UV-absorbance of tryptophan at 280 nm. The filtrate (free) peptide amount was corrected with the peptide loss during the filtration process by a control experiment with defined peptide amount.

### *In vitro* experiments

*In vitro* toxicity spectroscopy studies were performed using Glomax Multi+ detection system (Promega, Madison, WI, USA). Micrographs were performed on a Leica DMI6000 B with IMC in connection with a Leica DFC360 FX camera and AF 6000 software (Leica Microsystems, Vienna, Austria).

### PI-uptake assay-spectroscopy

Cells were collected, resuspended in respective media (see cell culture) and diluted to a concentration of 10^6^ cells/ml. Aliquots of 10^5^ cells were incubated in presence of respective concentrations of peptide (0 μM up to 100 μM) for up to 8 hours at 37°C and 5% CO_2_. Excitation and emission wavelengths were 536 nm and 617 nm, respectively. Cytotoxicity was calculated as described previously [6, 9].

### PI-uptake assay-fluorescence microscopy

Cells (1-5×10^4^) were seeded on Ibidi μ-Slide 8 wells and grown in 300 μl respective media for 2-3 days to a confluent layer. Propidium iodide (PI, 2 μl of 50 μg/ml in PBS, Biosource, Camarillo, CA, USA) was added to the well and cell status was checked after 5 minutes of incubation in the dark at room temperature. Then, peptide was added to the desired concentration and peptide effect was followed immediately. Pictures were taken every 5 or 15 minutes for up to 8 hours from the same section of cells. Excitation and emission wavelength were as follows: PI excitation, 535 nm and emission, 617 nm.

**MTS viability assay-spectroscopy**—Cell proliferation was measured by using CellTiter 96® Aqueous One Solution Cell Proliferation assay (Promega). Cells were plated in 96-well plates and grown until confluence in respective medium containing 2% serum at maximum to avoid interference with the MTS compound. Peptides were added to a final concentration of 5-100 μM. After incubation for 24 h at 37°C (5% CO_2_) MTS [3-(4,5-dimethylthiazol-2yl)-5-(3-carboxymethoxyphenyl)-2-(4-sulfophenyl)-2H-tetrazolium]-phenozine methosulfate solution (20 μl/well) was added and cells were again incubated for 2 hours at 37°C (5% CO_2_). The MTS compound is bio-reduced by cells into a colored formazan product that is soluble in tissue culture medium. The quantity of the formazan product as measured by the amount of 490 nm absorbance is directly proportional to the number of living cells in culture. Data are calculated as a percentage of the control (untreated) samples and represent the average of three wells in one experiment which was repeated three times per cell line.

### *In vivo* experiments

20 FOXN1 mice (Charles River Laboratories, Sulzfeld, Germany) were xenotransplanted with melanoma cells A375 subcutaneously in the right flank. The tumor was grown to a maximum length and width of 3 mm before peptide treatment was started (day 0). 9 mice were treated with peptide dissolved in buffer PBS (P), 5 mice were treated with pure buffer (C+), 5 mice were untreated (C-). Treatment occurred at days 0, 1, 6, 7, 8, 11 and 12 in 7 single doses by injection into the tumor. The total dose per mouse was 1.67 mg. The single doses applied at day 0 and 1 contained 0.33 mg peptide and the residual doses contained 0.20 mg. The tumor sizes were measured with manual calipers during the treatment time span. At day 15 the mice were sacrificed and the tumors were isolated and examined together with the organs of the mice after paraffin embedment and staining of four-micron sections with hematoxylin and eosin. All animal work was done in accordance with the protocol approved by the institutional animal care and use committee at the Austrian Federal Ministry for Science and Research (BMWF) (vote 66.010/0160-II/3b/2012).

The tumor sections of peptide treated and untreated mice were evaluated for the following: tumor cell types (spindle, epithelioid, or mixed), tumor size, present/ absent necrosis or fibrosis. Immunohistochemical studies were performed in all cases to evaluate the proliferative activity. We performed Ki67 staining (Ki-67 rabbit primary antibody, 30-9; ready to use with Ventana iView DAB detection kit; Pretreatment: CC1mild; All Ventana Medical Systems, Inc, Tucson, Arizona).

### Cell lines and abbreviations

A375, human malignant melanoma cell line; NHDF, normal human dermal fibroblasts; MUG Mel1, Medical University Graz human melanoma metastases to the brain; SBcl-2, human melanoma cell line; WM164, human melanoma metastases; ANTS, 8-aminonaphthalene-1,3,6-trisulfonic acid; BSA, bovine serum albumin; CD, circular dichroism; CHCl_3_/CH_3_OH, chloroform/methanol; CL, cardiolipin; DPPC, 1,2-dipalmitoyl-sn-glycero-3-phosphocholine; DPPS, 1,2-dipalmitoyl-sn-glycero-3-phospho-L-serine (Na-salt); DPX, p-xylene-bis-pyridinium bromide; hLFcin, human lactoferricin; FBS, fetal bovine serum; LF11, 11 amino acid fragment of hLFcin; LUV, large unilamellar vesicle; PBS or NaPi, sodium phosphate buffer; bLFcin, bovine lactoferricin; POPS, 1-palmitoyl-2-oleoyl-*sn*-glycero-3-phospho-L-serine; POPC, 1-palmitoyl-2-oleoyl-sn-glycero-3-phosphocholine; PI, Propidium iodide; PS, phosphatidylserine; R-DIM-P-LF11-334, Retro-dimer-proline-LF11-334 (= LF11-334-Proline-LF11-334 retro).

### Statistics

All values were presented as the mean ± SEM. *In vivo* data were analyzed by using the unpaired student's t test, p-values less than 0.001 were considered statistically significant.

## CONCLUSIONS

The peptide R-DIM-P-LF11-334, derived from human host defense, showed strong activity against melanoma and even their metastases. Especially effectivity against so far not treatable brain melanoma metastases (MUG Mel1), 24 hours stability in presence of blood components and a potential ability to pass the blood brain barrier are of great advantage for a potential therapy. Further toxicity against non-cancer cells is negligible at concentrations exhibiting high toxicity against cancer cells. In that respect cancer toxicity of the peptide against melanoma or their metastases was 20- to 25-fold compared with its activity against “non-cancer” cells. Supported by model studies the peptide seems to enter the cell via the PS “key” and probably localize within the cell where it induces slow killing by apoptosis. This was also shown to be the killing mechanism of the related peptide R-DIM-P-LF11-322. Of great impact for a clinical development of the peptide are the results obtained from a mouse tumor model of human melanoma that demonstrated that the peptide is not only able to inhibit tumor growth in cell culture but also in *in vivo* in mouse xenografts. After peptide treatment the tumors showed strong regression or even complete disappearance.

Although within this study the focus was on human malignant melanoma, a cancer hardly treatable and so far with poor prognosis, application of this peptide for various cancers is feasible, since the specific target PS has been shown to be present on cancer surfaces independent on type.

## SUPPLEMENTARY MATERIALS FIGURE AND TABLE


