# Supplementary material for: In vitro and in vivo cytotoxic activity of human lactoferricin derived antitumor peptide R-DIM-P-LF11-334 on human malignant melanoma
Source: Oncotarget. 2017 May 11;8(42):71817–32. doi: 10.18632/oncotarget.17823 (PMC5641092; doi:10.18632/oncotarget.17823)
Supplement: Supplementary file 1 [file oncotarget-08-71817-s001.pdf]

In vitro and *in vivo* cytotoxic activity of human lactoferricin derived antitumor peptide R-DIM-P-LF11-334 on human malignant melanoma

SUPPLEMENTARY MATERIALS

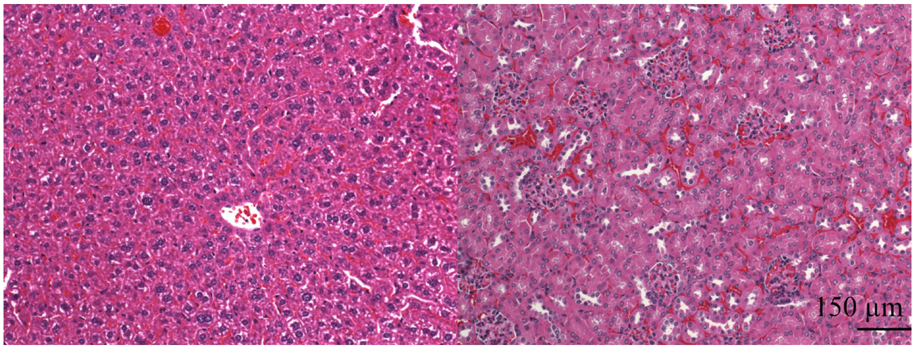

Supplementary Figure 1: No effect of peptide treatment on organs of mice. Tumor sections of liver (left) and kidney (right) stained with hematoxylin and eosin are shown. Mice had been treated with 9 doses of 0.68 mg peptide R-DIM-P-LF11-334 per mouse subcutaneously. After the last dose mice were sacrificed, organs were embedded and sections were stained with H&E. No effect of the peptides in liver and kidney were observed.

Supplementary Table 1: Comparison of median blood levels of mice xenografts of control with buffer PBS or peptide treated

	C+	+ P	Normal range
**Leukocytes**			
LEU [10^3/µl]	**10.25** ↑	6.13	4-10
Lym [%]	74.93	**82.28**↑	70-81
Mon [%]	4.83	4.46	1-5
N/Gr [%]	8.96	**8.96**↓	10-30
Eos [%]	0.23	1.33	<10
Bas [%]	0.63	0.68	
Other [%]	3.77	2.30	
**Erythrocytes**			
ERY [[10^6/µl]	9.07	8.53	6-11
MCV [fl]	**52.77**↑	50.18	35-50
Hct [%]	**47.80**↑	42.81	35-45
MCH [mg]	17.47	16.48	13-22
MCHC [g/dl]	33.27	32.85	24-40
Hb [g/dl]	15.80	14.06	14-18
EVB-SD [fl]	37.13	35.95	
EVB-CV [%]	19.17	19.44	
µERY [%]	10.37	16.00	
MERY [%]	0.63	0.34	
**Thrombocytes**			
THR [10^3/µl]	**650.00**↑	**705.00**↑	100-600
MPV [fl]	5.93	5.91	3-8
TKT [%]	0.40	0.43	
Mod [fl]	2.73	3.05	
Medn [fl]	4.33	4.14	
TVB	9.50	10.10	6-10
µTHR [%]	15.77	16.54	
MTHR [%]	5.90	8.13	

Average was determined from 4 control mice with buffer PBS (C+) and 7 mice treated with peptide (+P).
